# Bioluminescence imaging: a shining future for cardiac regeneration

**DOI:** 10.1111/jcmm.12018

**Published:** 2013-02-12

**Authors:** Santiago Roura, Carolina Gálvez-Montón, Antoni Bayes-Genis

**Affiliations:** a ICREC Research Program, Fundació Institut d′Investigació en Ciències de la Salut Germans Trias i Pujol (IGTP)Badalona, Spain; b Cardiology Service, Hospital Universitari Germans Trias i PujolBadalona, Spain; c Department of Medicine, Universitat Autònoma de BarcelonaBarcelona, Spain

**Keywords:** Bioluminescence, photoprotein, luciferase, bioanalysis, cell monitoring, cardiac regeneration

## Abstract

Advances in bioanalytical techniques have become crucial for both basic research and medical practice. One example, bioluminescence imaging (BLI), is based on the application of natural reactants with light-emitting capabilities (photoproteins and luciferases) isolated from a widespread group of organisms. The main challenges in cardiac regeneration remain unresolved, but a vast number of studies have harnessed BLI with the discovery of aequorin and green fluorescent proteins. First described in the luminous hydromedusan *Aequorea victoria* in the early 1960s, bioluminescent proteins have greatly contributed to the design and initiation of ongoing cell-based clinical trials on cardiovascular diseases. In conjunction with advances in reporter gene technology, BLI provides valuable information about the location and functional status of regenerative cells implanted into numerous animal models of disease. The purpose of this review was to present the great potential of BLI, among other existing imaging modalities, to refine effectiveness and underlying mechanisms of cardiac cell therapy. We recount the first discovery of natural primary compounds with light-emitting capabilities, and follow their applications to bioanalysis. We also illustrate insights and perspectives on BLI to illuminate current efforts in cardiac regeneration, where the future is bright.

- Introduction
- More than a century of bioluminescence: description of its molecular basis
- Application of light-emitting proteins to bioanalysis
- Imaging modalities in cell-based cardiac therapy
- Luminescent expression reporters illuminate cardiac regeneration efforts
- Conclusions and future perspectives

## Introduction

**Fig. 1:**
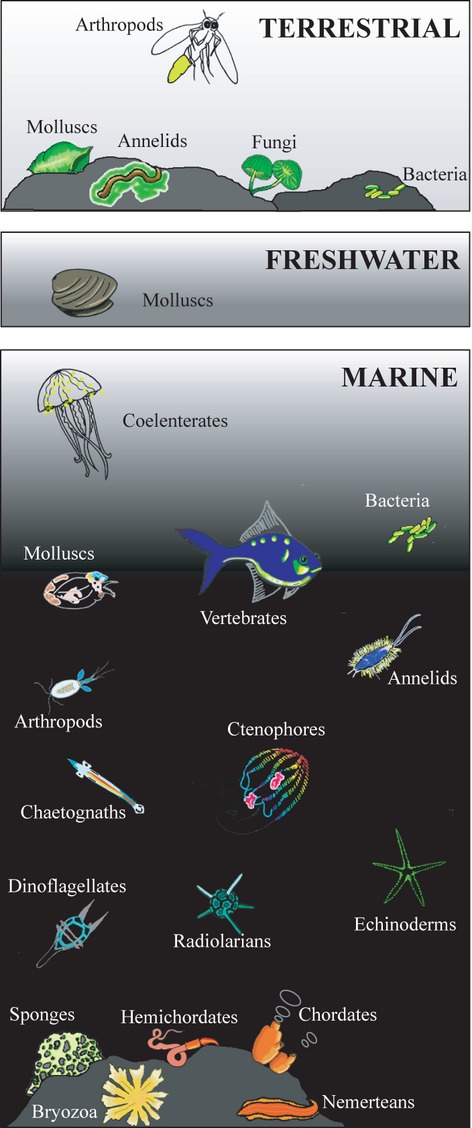
Global ‘bioluminescent’ biomes. Rarely on land, but widely common in deep-seas and oceans, a vast number of creatures has been discovered as visible light-emitting organisms.

Bioluminescence is an amazing natural phenomenon, where light is produced by an organism; *e.g*. on summer nights, different species of terrestrial Lampyridae beetles, popularly known as fireflies, emit ‘flashes of light’ to find each other over long distances 1. This reaction also occurs in the luminous organs of a variety of deep-sea organisms to survive and communicate where sunlight is weak or absent; to camouflage; to blind aggressors; to escape from predators; and to refine the attack 2, 3. Thus, it is estimated that bioluminescent reactions occur in numerous organisms that are widespread in nature (Fig. 1) 4. The mechanisms that give rise to bioluminescence photons are different from those that cause light emission from other sources, like the sun or a light bulb, where energy arises from heat. Mostly, this light is generated by internal reactants, called photoproteins and luciferases. However, to date, only a few bioluminescent proteins have been studied in detail.

Stem cell–based therapies represent a promising treatment for cardiac damage, like that due to myocardial infarction. However, complete functional restoration has remained unsuccessful, despite intensive research in recent years in both experimental and clinical contexts 5–9. Problematically, survival of implanted cells within diseased tissues remains as one of the main concerns prompting valuable information to correlate therapeutic outcome and cell persistence, as well as to identify the most suitable cell type and dose.

Application of distinct imaging modalities to overcome cardiac regeneration hurdles is basically at a developmental stage in animal models 9–14; *e.g*. magnetic resonance imaging (MRI) provides precise spatial resolution, whereas bioluminescence imaging (BLI) easily reports location and functional status. By using BLI, the activation of lineage-specific promoters driving the expression of photoproteins or luciferases can be used to track implanted cell behaviour. To date, BLI has been particularly useful *in vitro* where bioluminescence emission correlates linearly with emitting cell density, and in small animal models because there is limited loss or attenuation of a luminous signal due to the scattering phenomena when light must transmit across dense body tissues 15.

The purpose of this review was to present the great potential of BLI, among other existing imaging modalities, to refine effectiveness and underlying mechanisms of cardiac cell therapy before its clinical approach; we recount the first discovery of natural primary compounds with light-emitting capabilities and follow their applications to bioanalysis. We also provide insights and perspectives encouraging researchers to harness BLI to illuminate their current intensive efforts in cardiac regeneration.

## More than a century of bioluminescence: description of its molecular basis

The first historical references to light-emitting reactions, including the introduction of the terms luciferin and luciferase, dated from 1885, when Emil du Bois-Reymond (1818–1896, Berlin) mixed two different extracts from clams and beetles and produced light. This German scientist also found that one of the extracts was heat-sensitive, which led to the conclusion that there were at least two components in the reaction. The heat-sensitive chemical was hypothesized to be an enzyme, which he called luciferase; the heat-resistant compound was referred to as luciferin (from the Latin Lucifer, ‘Light-bringer’).

In recent history, numerous significant advances have been made in the biochemistry of bioluminescence 16, 17. Briefly, in 1947, it was recognized that ATP (prepared from rabbit muscle) was the energy source for *in vitro* light-emitting systems. Next, Strehler and Totter were the first to study the firefly reaction, which was not fully described until 13 years later. The firefly luciferase and luciferin structures were subsequently resolved by McElroy *et al* in 1956 and 1961 respectively.

**Fig. 2:**
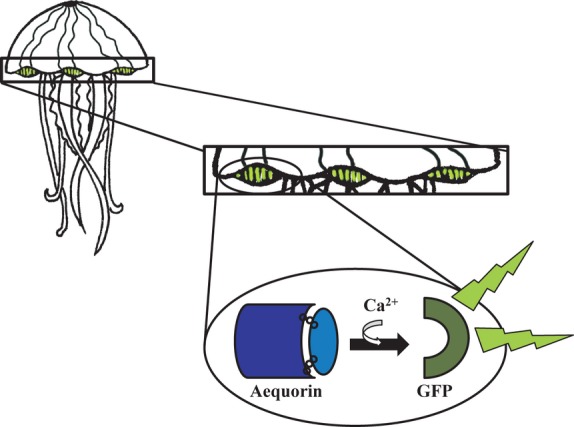
*Aequorea victoria* bioluminescence. Ca^2+^-activated aequorin and accessory GFP naturally coexist in the luminous organs of the crystal jellyfish. When stimulated, photocytes distributed along the edge of the umbrella give off green light due to a radiationless energy transfer (black arrow) from aequorin to GFP. This phenomenon has allowed the development of the novel hybrid bioluminescence FRET techniques, based on the combination (protein fusion) of photoproteins or luciferases and fluorescent proteins, to visualize protein–protein interactions inside single living cells and animals. Abbreviations: Ca^2+^, calcium; GFP, green fluorescent protein.

It was not until the early 1960s that the first bioluminescent proteins were purified from the luminous hydromedusan *Aequorea victoria*, which produces a blue light. First, aequorin (blue light emitter) was discovered by Shimomura *et al* and, later, they also described the green fluorescent protein (GFP), which takes the blue light and shifts it to a green colour (Fig. 2). These discoveries opened a new area in the study and application of BLI 18–20. Originally, aequorin was shown to have the ability to emit light in aqueous solutions by merely adding a trace of Ca^2+^ in the absence of oxygen 21. At that time, despite corroboration that light was emitted following an intramolecular reaction inside the protein molecule, aequorin was thought to be an exceptional protein that was an accident of nature. However, in 1966, another protein that emitted light when a peroxide and a trace of Fe^2+^ were added in the presence of oxygen was discovered in the parchment of the Chatopterus worm 22. Because this protein also produced light without the participation of an enzyme, Shimomura and Johnson coined the term ‘photoprotein’ to designate these light-emitting proteins that do not fit the classical foundation by which an enzyme (luciferase) catalyses the oxidation of a smaller organic substrate molecule (luciferin) with light emission. Thereafter, further luciferases and photoproteins were brought into the light 23–26.

Regarding the molecular basis of the bioluminescence reaction, the term ‘photoprotein’ refers to a primary reactant found in a specialized organ of an organism that is capable of emitting light in proportion to the amount of protein (rather than, in this case, to the amount of a theoretical or non-existing substrate), and that is not the unstable, transient intermediate of a characteristic enzyme–substrate reaction 27. Photoproteins are molecular complexes consisting of a first compound called coelenterazine, apo-photoprotein and molecular oxygen. When bioluminescence reaction is triggered, a second compound (singlet-excited coelenteramide) is generated from the oxygen-preactivated coelenterazine and light is produced during its decay to the ground state 28. In contrast, luciferases act in a two-step reaction, as it was described for the first time studying the North American firefly *Photinus pyralis*, one of the most well-studied among all the bioluminescent organisms 29, 30. Studies on the firefly luciferase (FLuc or PLuc) revealed that first the luciferase substrate, luciferin, reacts with ATP-Mg^2+^ to generate inorganic pyrophosphate and an intermediate luciferyl-adenylate; second, this transient intermediate is oxidized and decarboxylated to form oxyluciferin, the light emitter, which produces CO_2_, AMP and photons of yellow-green light (ranging from 550 to 570 nm) when returns to the baseline conformation 31. Furthermore, the rapid increase in light output to a maximum (measured in relative light units) is followed by a progressive decline in the emission intensity 32. In the luciferase–luciferin reaction, the total amount of light emitted is proportional to the amount of luciferin (substrate), not to the amount of luciferase (enzyme). The chemical structures of the reaction compounds also play a significant role on the stability of luciferases, and they specify the colour of emitted light 33, 34. In addition to luciferin, oxygen and luciferase, other cofactors are required for bioluminescent reactions such as Ca^2+^, Mg^2+^ and ATP.

Other important milestones in this field included: the elucidation of the mechanism of bioluminescence in many marine organisms (1967) 35; a technical report that described the use of a luminescent bacterial system for rapid assessment of aquatic toxicity, which provided the basis for the first patent in the application of bioluminescence methodology (1981) 36; and a description of the primary structure of the GFP 37.

## Application of light-emitting proteins to bioanalysis

Current research endeavours have increasingly demanded fast, portable, easy-to-use bioanalytical methods with a high level of functional integration. Bioluminescent reactants are quite versatile, sensitive tools with many attractive properties. They can be detected at extremely low concentrations; they avoid background interference from autofluorescent compounds typically present in biological samples; and they are compatible with many miniaturized platforms, like the lab-on-a-chip and lab-on-a-CD platforms. Thus, some of the above-mentioned light-emitting molecules have been used in a plethora of assays, including: intracellular assays for monitoring important biological molecules, like ATP and calcium; genetic assays involving regulation and detection; immunoassays; binding assays; and whole-cell biosensor assays, among others 38, 39.

One of the first applications of both photoproteins and luciferases, particularly the Ca^2+^-sensitive forms derived from coelenterates and jellyfish, was to measure and monitor calcium ions within various biological systems, mainly single cells 40, 41. These pioneering studies provided the basis for the design and development, only few years later, of novel methods for incorporating the obelin apoprotein and mRNA isolated from the hydroid, Obelia, into the cytoplasm of intact human neutrophils. This approach avoided the consumption of Ca^2+^-activated photoproteins during cell activation or injury and provided a means to monitor protein synthesis in living cells 42.

**Fig. 3:**
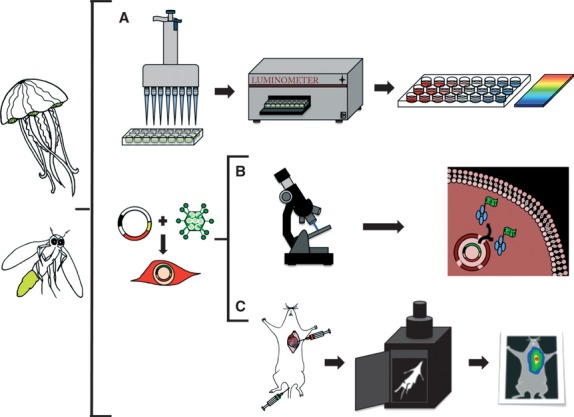
Schematic representations of major BLI applications. Bioluminescence-based assays are being currently applied for (**A**) detection of biological molecules, (**B**) gene/protein expression and intracellular trafficking, and (**C**) implanted cell distribution and function.

Subsequent bioluminescent analytical applications included quantifications of ATP 43–45 and both the non-reduced and reduced forms of nicotinamide adenine dinucleotide (NADH) 46, 47. Taken together, these investigations provided the basis for the design and development of a broad repertoire of reactions in the laboratory setting. These included the forensic luminol test (where traces of blood at crime scenes could be detected upon contact with the iron in haemoglobin) 48, the cell-surface-localized ATP detection assay 49, and bioluminescent assays for high-throughput screening (Fig. 3A) 50.

The GFP, together with other fluorescent proteins with a wide colour range of emission (from blue to far-red) and versatile biochemical and photophysical properties, has also revolutionized the applications of molecular biology 51. As a result, over 150 different fluorescent GFP-like proteins have been described in marine organisms (none in terrestrial organisms) and divided into seven groups according to their colour and chromophore structure 52.

For example, it was initially recognized that protein labelling was feasible by producing chimeric proteins fused to GFP 53, 54. This use has greatly extended our understanding of protein dynamics, including protein movements and interactions (Fig. 3B). Subsequently, advances in gene technology provided inducible reporters and viral constitutive reporters that were integrated with GFP in fusion proteins to study intracellular Ca^2+^ activity, monitor gene expression and track cells in intact living organisms 55. For example, a GFP labelling system was used to validate the hypothesis that human bone marrow–derived mesenchymal stem cells (MSC) possessed tropism for brain tumours, and thus, they could be used as vehicles for delivering glioma therapies 56. Novel molecular chimeras have been recently designed, including GFP or monomeric red fluorescent protein (mRFP) fused with aequorin, or *Vibrio fischeri* Y1 yellow fluorescence protein fused with bacterial luciferase hydroxyflavin. These chimeric proteins have yielded improved bioluminescent Ca^2+^ sensors for detecting intracellular Ca^2+^ changes in single cells, like neurons, zebrafish and mice 57–61. Recently, the colour palette available with the *Aequorea victoria* jellyfish photoproteins (blue to yellow) was expanded to include other monomeric fluorescent proteins (orange to far-red) derived from non-luminous Anthozoa (corals and sea anemones). In addition, new optical highlighters and Förster Resonance Energy Transfer (FRET) biosensors were developed. Thus, with these tools, we predict that any biological parameter can be investigated with an appropriate fluorescent protein–based application 62–64.

The harnessing of animals for research has been crucial for numerous medical advancements, including the development of effective treatments and the understanding of mechanisms involved in human disorders. Experimental procedures for testing regeneration in animal models typically require accurate characterization of implanted cell behaviour and functional benefits, including cell distribution, survival and differentiation status. Many existing analysis techniques are destructive; this necessitates replicating the experiments. Alternatively, bioluminescence offers the potential for non-destructive monitoring (Fig. 3C); moreover, the development of instrumentation for detecting weak optical signals, like cooled, sensitive charge-coupled device (CCD) cameras 65, has reduced the number of repetitions required. This technique also allows continuous detection and quantification of information minimizing the necessary number of experimentation animals according to the 3Rs (replacement, reduction and refinement) principle 66. Furthermore, despite the apparent opacity of tissues, light can also be used to examine physiological functions and anatomic structures at depths of several millimetres in live animals 67.

The first photonic detection in small mammals was performed in mice infected with bacteria that expressed luciferase proteins from a set of genes, the lux operon, which encoded the self-regulation and production of luminescent proteins 68. Lately, it was shown that the human immunodeficiency virus promoter fused to firefly luciferase allowed real-time, external monitoring of gene expression, both superficially and in deep tissues in mice 69. Remarkably, images were also obtained showing the dynamics of neoplastic cells labelled with luciferase expression 70–72. When an optimized version of RLuc was functionally expressed in mammalian cells 73, it became possible to design dual labelling approaches with RLuc and FLuc coexpression. This strategy was used to show that neural precursor cells that over-expressed a secreted form of tumour necrosis factor-related apoptosis-inducing ligand could migrate into glioblastomas and selectively kill neoplastic cells 74. Recently, this approach was also applied to simultaneously monitor the proliferation and chondrogenic differentiation of human adipose tissue–derived stromal cells 75.

The above-mentioned studies collectively exemplify the progress made in non-invasive imaging. This research has added new dimensions to our comprehension of several human diseases, including infection progression 76–78 and cancer. With these approaches, we can evaluate tumour growth and metastases and track cell-mediated treatments 79–82. In addition, we can detect the induction of proteins by specific stimuli 83.

## Imaging modalities in cell-based cardiac therapy

There are fundamentally three advancing non-invasive cell-tracking methods contributing to cardiac regeneration and translation to clinic: (1) paramagnetic labelling for MRI; (2) radioactive labelling for nuclear imaging, including positron emission tomography (PET) and single photon emission computed tomography (SPECT); and (3) reporter gene transduction for MRI, PET, SPECT, fluorescence and bioluminescence imaging 84, 85. For MRI, contrast agents such as those most widely employed based on gadolinium are mandatory to provide distinct signal intensity to labelled cells from background 84. Cells can be also enriched with supermagnetic iron oxide (SPIO) particles detected at the micromolar level. Due to its characteristic high resolution together with recent advances in visualization of molecular pathways, MRI is one of the most effective techniques for diagnosis and to translate preclinical results to clinical practice. In contrast, gamma ray–emitting radionuclides or radioisotopes are also advantageous to visualize *in vivo* cell trafficking due to low background signal and high signal-to-noise ratio, but permit low spatial resolution.

Powerful cost-efficient optical imaging systems allow alternative high-throughput spatial and temporal monitoring with no radioactivity in the preclinical context. These exciting methodologies are based on the transfer of reporter genes, typically either bioluminescent or fluorescent, into cells. Successful reporter gene expression, which only occurs in viable modified cell, can be evaluated for quality control before *in vivo* implantation. Imaging of cells carrying reporter genes also holds promise for studying the subcellular basis of cell maturation and differentiation because they may be regulated under restrictive and lineage-specific promoters. In addition, multiple combinations of reporter genes can be applied into the same cell.

Although the choice will depend on the intrinsic properties of each molecular-functional imaging tool, including availability and cost-benefit relationship as well as the biological process analysed, cumulative experience provides some foundations: (1) while MRI shows high resolution suffering from relatively low detection sensitivity, PET has both great sensitivity and resolution, but requires high-cost facilities; (2) bioluminescence and fluorescence procedures have excellent sensitivity, but relative depth penetration 86; (3) reporter gene–based imaging can be performed repeatedly in contrast to direct cell labelling which is limited by radioactivity decline; (4) BLI is more sensitive than fluorescence, despite the latter does not need substrate injection and may be applied when sensitivity is not as decisive 87; (5) gadolinium-based contrast has the risk of side effects 88; (6) BLI has some hurdles (including light attenuation through large animal tissues and inability to differentiate superimposed anatomical structures) that are beginning to be solved by three-dimensional reconstructions 89; and (7) there are objections against human genetic modification that preclude the clinical use of BLI until development of DNA-free expression procedures.

## Luminescent expression reporters illuminate cardiac regeneration efforts

Ischaemic heart failure is the end stage of major cardiovascular diseases. It most often occurs when the blood supply to the heart is blocked. This leads to myocardial ischaemia and necrosis following the formation of a non-contractile scar and subsequent ventricular remodelling 5. Current heart failure strategies include pharmacology or surgical interventions, but the only definitive treatment is heart transplantation. Heart transplants are limited by the scarcity of donors and by graft rejection over time.

**Fig. 4:**
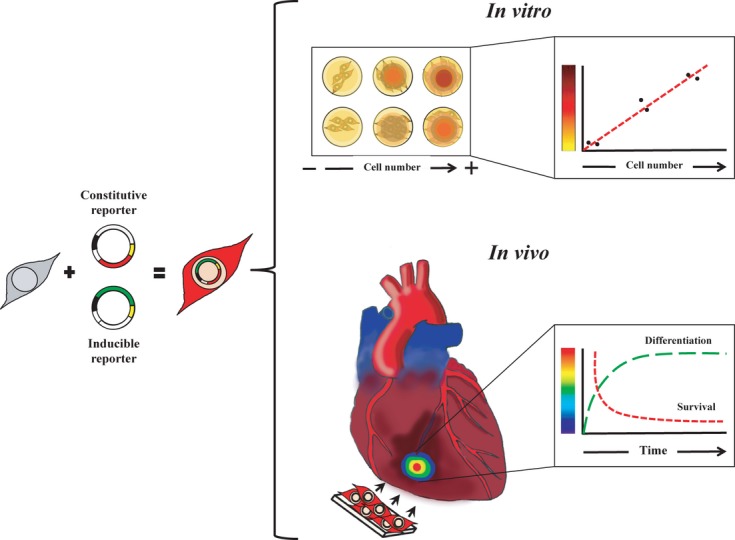
Harnessing of BLI in cardiac regeneration. BLI is supported by advanced gene technology that allows the generation of efficient reporters comprising genes encoding for distinct luciferases under the control of constitutive and inducible, lineage-specific promoters. This approach is first useful *in vitro* to modify therapeutic cells, to validate, *e.g*. for duplicate in multi-well culture plates, the increase in photonic detection levels with increasing cell number, and, lately *in vivo*, to track at any given time implanted cell survival and differentiation. As a result, researchers can finely quantify cardiac regeneration degree relative to the number of surviving cells under ischemic conditions.

**Fig. 5:**
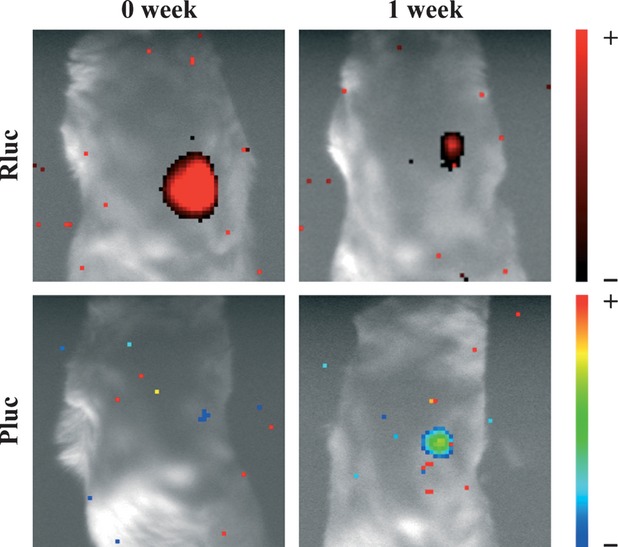
Example of non-invasive BLI monitoring of cardiac differentiation in the experimental model of acute myocardial infarction in mice. Representative BLI images showing decrease in the activity of RLuc driven by the constitutive CMV promoter, but increase in that from PLuc under the control of the cardiac-specific cTnI promoter within adipose tissue–derived progenitor cells at indicated times after myocardial implantation.

Stem cell–based therapies are a promising strategy for regeneration of injured cardiac tissue, partially contributing to the generation of new myocardial tissue and vessels 6–9. Nevertheless, despite progressive improvements, the best cell type and delivery strategy are not well-established 8, 90–95. The need to optimize cell survival and resolve implantation difficulties due to adverse mechanical forces and hypoxia 96 has motivated the emergence of new approaches in this field, both for cell delivery (by cell injection or bioengineered implants) and for implanted cell detection(Fig. 4). BLI was first used to track engraftment, survival and rejection of transplanted tissues, including cardiac allografts, in luciferase-expressing transgenic mouse 97. As a result, this imaging approach was seen to be sensitive, reproducible and valuable widely across cardiac regeneration approaches such for the study of the dynamic range of the entire process of cell transplantation. Subsequently, it was the first published study to report broad applications of this versatile imaging platform on stem cell therapy monitoring embryonic stem cell survival, proliferation and migration after cardiac delivery 98. Following cardiac transplantation of adipose tissue- and bone marrow-derived MSCs with the β-actin promoter driving FLuc and GFP expression, BLI showed drastic administered cell loss within 4–5 weeks post-implantation 99. Bai *et al* also studied the delivery of both cultured and freshly isolated adipose tissue–derived stem cells modified to co-express FLuc and GFP reporter genes following acute myocardial infarction in severe combined immunodeficient mice 100. Importantly, neither cell transduction (mainly using lentivirus) nor FLuc/GFP expression did change proliferation rate and differentiation potential of pre-implanted cells 101. Moreover, Hung *et al*. used luciferase-based imaging to assess variable embryonic stem cell viability after transplantation into infarcted mice 102. These authors applied a fibrin glue to improve cell engraftment and to increase the persistence of different regenerative cell types in the post-infarcted myocardium. Enhanced co-delivery of human embryonic stem cell–derived vascular and smooth muscle cells 103 modified to express GFP and FLuc 104 was confirmed by BLI both *in vivo* and *ex vivo* in mice and pigs respectively. In both models, meaningful functional benefits were also reported 105. Another BLI study was based on the use of two distinct luciferase-engineered reporters: one carrying the tissue-specific cardiac troponin I promoter and another comprising the constitutively expressed cytomegalovirus (CMV) promoter regulating the expression of PLuc and RLuc respectively. This strategy allowed the simultaneous monitoring of cell survival and cardiomyogenic differentiation of cardiac adipose tissue–derived progenitor cells *in vivo* 106 (Fig. 5). Similarly, a cardiac sodium–calcium exchanger-(NCX)-1 promoter-regulated FLuc was used to demonstrate efficient cell engraftment and differentiation in the background of a mouse embryonic stem cell line, also carrying the enhanced yellow fluorescent protein, in transgenic mice over-expressing adenylyl cyclase 6 and with reduced left ventricular fibrosis 107, 108.

Basically, cell implantation approaches have included intracoronary delivery, direct intramyocardial injection or endomyocardial injection using specifically designed catheters 106. More recently, as these delivery procedures have showed modest outcome in cardiac function recovery and limited cell survival in the fibrous myocardium 109, it is being developing the promising biomedical technology for the treatment and replacement of injured tissues (including infarcted myocardium), otherwise known as tissue engineering, in which BLI monitoring constitutes a helpful tool together with other pillar principles from materials science, cell biology, transplantation and engineering 103, 106, 108, 110–112.

Endothelial recovery is also a key challenge in cardiac regenerative medicine 113. Many regenerative studies attribute functional improvements to increased myocardial neovascularization typically found in cell-treated animals 100, 101, 105, 114. In this context, BLI is also a helpful approach for studying the expression of key regulatory vascular genes and the differentiation of implanted cells into the endothelial lineage. For instance, a luciferase fused to vascular endothelial growth factor receptor (VEGFR)-2 was used to monitor and quantify VEGFR-2 expression and to study drugs affecting angiogenesis *in vivo* 115. In addition to bone marrow- and adipose tissue–derived cells 116–118, cells isolated from umbilical cord blood (UCB) have been shown to acquire phenotypic and functional characteristics of the endothelial lineage 119. Thus, UCB is considered to be an emerging, valuable stem cell source 120. In particular, BLI was used to follow the expression of chimeric luciferase/fluorescent proteins by UCB-derived MSCs in a mouse model of angiogenesis 119. The results showed that a fusion reporter vector comprising both PLuc and enhanced GFP under the transcriptional control of the human endothelial cell marker CD31 promoter was highly activated within injected UCBMSCs supporting efficient cell differentiation. Remarkably, in this study, injected cells also carried another chimeric reporter of ‘cell number’ comprising RLuc and mRFP under the control of the CMV promoter to evaluate cell differentiation degree independently of the number of cells. Once again, BLI in conjunction with advanced reporter gene technology allowed researchers to efficiently track cell behaviour in cardiac cell–based therapies using the ratio of the light produced by distinct luciferases and fluorescent proteins regulated by inducible (lineage-specific) and constitutive promoters (Fig. 4). More recently, these same authors employed these genetically modified UCBMSCs embedded in a fibrin patch to assess the effect of their implantation following acute myocardial infarction 121.

Taken together, this experience demonstrates that BLI is a valuable, unpolluted tool for the analysis of cardiac regeneration, will extend our understanding of the basis of optimal cell administration system and/or dose, and will greatly influence our views on the efficacy of future cell-based therapies. In comparison with other imaging modalities that also maintain cell viability, phenotype and differentiation capacity *in vitro*, luciferase-based cell labelling is not associated, *e.g*. with potential release of cytotoxic or pro-inflammatory particles when cells labelled with SPIO for MRI are being removed *in vivo* 122.

## Conclusions and future perspectives

Although the evolutionary origins of bioluminescence remain obscure, light-emitting reactions include ecologically functional communication, courtship, attack-defensive and camouflage tools 3, 123. Over the past decades, advances in bioimaging techniques have become crucial for both basic research and medical practice 124. Although many challenges in cardiac regeneration remain unresolved, the vast number of studies that have employed bioluminescence have undoubtedly contributed to the design and initiation of ongoing cell-based clinical trials 125.

From the first description of light-emitting aequorin and of its accessory protein GFP in the jellyfish *Aequorea victoria* 126, there has been an exponential increase in practical applications for bioluminescence. These include a wide range of analytical assays for studying important biological metabolites such as ATP and Ca^2+^ 127. Undoubtedly, much of what is now known and widely accepted about bioluminescence and its molecular basis has been derived from the pioneer studies in *Aequorea victoria*. Thus, these revolutionary discoveries were deservedly recognized in 2008, when Shimomura, Chalfie and Tsien were jointly awarded with the Nobel Prize in Chemistry for the discovery and development of GFP.

Finally, the advent of reporter gene technology has also received much attention, because the light produced by photoproteins, luciferases and chimeras can be followed in single cells or in whole living organisms. This approach has promoted studies in gene expression, disease progression, and drug- or cell-based treatments of human multifaceted diseases. Presently, the major drawback of BLI is that it remains clinically unavailable focusing on animal models of diseases. However, new approaches have emerged for introducing specific mutations into genes that encode bioluminescent proteins; this has generated novel light-emitting reactants with different colour emissions 128, 129, higher luminescence intensities 130 and improved thermostabilities 131 or catalytic efficiencies 132, 133. In BLI, diverse technological fields such as molecular biology, genetics, stem cell therapy and bioimaging converge; this is beginning to change the view of both researchers and clinicians carrying regenerative medicine, including cardiac regeneration, into a new era. Therefore, it is not unreasonable to envision that the future looks bright for cardiac regeneration.
